# Fat mobilization without weight loss is a potentially rapid response to nicotinamide riboside in obese people: it's time to test with exercise

**DOI:** 10.1093/ajcn/nqaa109

**Published:** 2020-05-15

**Authors:** Noah T Fluharty, Charles Brenner

**Affiliations:** Department of Biochemistry, University of Iowa, Iowa City, IA, USA; Department of Biochemistry, University of Iowa, Iowa City, IA, USA

See corresponding article on page 413.

Motivated to address the human obesity epidemic, molecular scientists have explored multiple interventions in mouse models. Like human office workers, laboratory mice have a limited latitude to move and thus experience weight gain when they consume food ad libitum. Unsurprisingly, mice exhibit greater and faster weight gain when they are provided high-fat diets (HFD). However, whereas common human obesity is established over decades of imbalance between energy intake and energy expenditure, mouse obesity can be induced and sometimes prevented or treated in 2 mo.

Four nicotinamide adenine dinucleotide (NAD) coenzymes, namely NAD^+^, NADH, NADP^+^, and NADPH, mediate the key transformations from food into energy, activity, biomass, and repair (1). The NAD metabolome is disturbed by several conditions of metabolic stress in multiple tissues. In the liver of overfed mice, NAD^+^, the key catalyst for fuel oxidation, is modestly depressed whereas levels of NADP^+^ and NADPH, the key catalysts for nucleic acid and lipid biosynthesis and control of reactive oxygen species, are greatly depressed (2).

Nicotinamide riboside (NR) is a precursor of NAD^+^ that acts through the nicotinamide ribose kinase pathway to replete the NAD metabolome (3) in a manner that is bioenergetically less costly than other routes to NAD (4). In male mice, NR blunts weight gain on HFD and simultaneously improves multiple metabolic parameters (2, 5). However, research has not established which are the driver and which are the passenger effects of boosting NAD in mice, whether any of these mechanisms are addressable in common human obesity and over what time frames, and whether there are sex differences in responses to NR.

For example, whereas mice on HFD — with or without low doses of streptozotocin to render them insulin insufficient — benefited from lower hepatic steatosis, lower circulating liver enzymes, better glycemic control, and preserved nerve structure and function when given oral NR (2), all of these effects could have been due to an effect on a nonmeasured parameter such as malabsorption or hyperactivity. Thus, if NR blunted weight gain due to shunting calories into waste or increased fuel oxidation due to higher physical activity, then the mice would obtain all of the physiological benefits of NR without direct modulation of insulin sensitivity. On the other hand, one or more key measured targets might be the driver effect that helped other metabolic parameters come into line. With the world clamoring for orally active obesity interventions and with human safety established for NR (6, 7), there has been a rush to test NR in people under conditions of metabolic stress in trials that are hopeful with respect to outcome but less than certain in the choice of primary endpoint.

Although there is no evidence that NR promotes malabsorption or hyperactivity, we could postulate many different mechanisms to account for why HFD mice did better on NR with primary targets as diverse as gluconeogenesis and behavior. In a perfect world, the mouse work would identify the proximal mechanism such that clinical trialists would know what endpoints to monitor and how to design the most sensitive trial. However, the metabolic syndrome is a complicated systemic problem in all vertebrates and the roles for NAD are highly pleiotropic, especially when one considers dozens of affected tissues and myriad disturbances in metabolism.

Because insulin sensitization and weight loss were the primary endpoints of the first human obesity trial, that trial could not randomize on any other parameter that might have been a driver (8). The trial might have incorporated physical activity coaching as a standard of care plus or minus NR, but it did not. Thus, it was not surprising that the first human obesity trial for NR showed excellent safety data but a top-line failure to promote weight loss in 12 wk in ∼105 kg ∼60-y old sedentary men. Poring through the data, however, we note the NR-supplemented group obtained a 2% reduction in their hepatic lipid content (from 11.3% to 9.3%) versus a 0.2% reduction in the placebo group (from 14.1% to 13.9%; *P* = 0.13 for the comparison between groups) (8).

Whereas the aforementioned trial used 2 g of NR/d for 12 wk in sedentary men (8), a new trial published in this issue of the *American Journal of Clinical Nutrition* deployed 1 g of NR/d for 6 wk in 13 overweight or obese men and women (9). The trial was placebo-controlled and featured a crossover design such that every participant had 6 wk of NR followed by 6 wk of placebo or vice versa. It will surprise few people familiar with the differences between 60-y old obese people and laboratory mice that the primary endpoint of increased insulin sensitivity was not met. However, the authors discovered that body composition was altered with a 1.34% reduction in the percentage of fat mass (*P* = 0.02) (9). The drop in fat mass was accompanied by an increase in fat-free mass (lean mass was not measured) and an increase in sleeping metabolic rate. Though increased metabolic rate would be expected with a gain in muscle mass and the sleeping metabolic rate divided by fat-free mass was not increased, the body composition improvement is a positive result and it was observed in 6 of 7 female participants. Though the trial was small and had a short time frame, it provided 2 easily measured biomarkers of NR-responsiveness: a change in body composition, which was measured by whole-body densitometry, and an increase in skeletal muscle acetylcarnitine (9). Blood glucose, inflammation, blood pressure, cardiac function, and magnetic resonance measures of liver and intramyocellular fat content were not affected by NR supplementation in this small trial. The participants in this study did not have elevated hepatic lipids, so it was not possible to expect confirmation of mobilization of hepatic lipids. However, coupled with a recent report that inflammatory biomarkers such as interleukin 6 are depressed by NR in older men in 3 wk (10), the clinical community is now discovering parameters that appear to respond quickly to NAD repletion.

We suggest that a depressed and/or metabolically challenged hepatic NAD metabolome is a potential target for NR interventions, and note that people with alcoholic liver disease have depressed hepatic NAD (11). It is clearly time to perform trials of longer duration, as well as ones that incorporate physical activity, to test the hypothesis that NR supports mobilization of fat from liver and other storage depots in exercised obese adults. If NR does indeed help people mobilize fat, increased physical activity should help people gain lean mass, increase resting metabolic rate, and eventually increase their exercise capacity in lifestyle-modifying ways that promote a virtuous cycle.

The human data may also have reverse translational value to dissect what happens in rodents. We suggest that NR initially mobilizes fat from the liver and, as the liver clears steatosis, glycemic control improves through a mechanism that does not involve direct insulin sensitization (2). Although improved glycemic control itself could potentially reduce neuropathy (2), NR provides resistance to chemotherapeutic neuropathy that has been clearly shown to have targets in the nerve (12, 13). Mechanistically oriented translational research that is cognizant of changes to tissue NAD metabolomes (14 ) and NAD gene expression programs will be required to identify patient populations potentially able to respond to NR supplementation.

## Notes

The authors’ contributions were as follows—NTF and CB: wrote the paper, and read, and approved the final manuscript.

Author disclosures: CB developed intellectual property on uses of nicotinamide riboside that have been developed by ChromaDex, Inc. He serves as chief scientific adviser for ChromaDex, owns ChromaDex stock and has received grant support from ChromaDex. NTF declares no conflicts of interest.

## References

[1] Belenky P, Bogan KL, Brenner C NAD+ metabolism in health and disease. Trends Biochem Sci. 2007;32(1):12–9. 17161604 10.1016/j.tibs.2006.11.006

[2] Trammell SA, Weidemann BJ, Chadda A, Yorek MS, Holmes A, Coppey LJ, Obrosov A, Kardon RH, Yorek MA, Brenner C Nicotinamide riboside opposes type 2 diabetes and neuropathy in mice. Sci Rep. 2016;6:26933. 27230286 10.1038/srep26933 PMC4882590

[3] Bieganowski P, Brenner C Discoveries of nicotinamide riboside as a nutrient and conserved NRK genes establish a Preiss-Handler independent route to NAD+ in fungi and humans. Cell. 2004;117(4):495–502. 15137942 10.1016/s0092-8674(04)00416-7

[4] Diguet N, Trammell SAJ, Tannous C, Deloux R, Piquereau J, Mougenot N, Gouge A, Gressette M, Manoury B, Blanc J et al. Nicotinamide riboside preserves cardiac function in a mouse model of dilated cardiomyopathy. Circulation. 2018;137:2256–73. 29217642 10.1161/CIRCULATIONAHA.116.026099 PMC6954688

[5] Canto C, Houtkooper RH, Pirinen E, Youn DY, Oosterveer MH, Cen Y, Fernandez-Marcos PJ, Yamamoto H, Andreux PA, Cettour-Rose P et al. The NAD(+) precursor nicotinamide riboside enhances oxidative metabolism and protects against high-fat diet-induced obesity. Cell Metab. 2012;15(6):838–47. 22682224 10.1016/j.cmet.2012.04.022 PMC3616313

[6] Conze DB, Brenner C, Kruger CL Safety and metabolism of long term administration of Niagen® (nicotinamide riboside chloride) in healthy overweight adults. Sci Rep. 2019;9:9772. 31278280 10.1038/s41598-019-46120-z PMC6611812

[7] Trammell SA, Schmidt MS, Weidemann BJ, Redpath P, Jaksch F, Dellinger RW, Li Z, Abel ED, Migaud ME, Brenner C Nicotinamide riboside is uniquely and orally bioavailable in mice and humans. Nat Commun. 2016;7:12948. 27721479 10.1038/ncomms12948 PMC5062546

[8] Dollerup OL, Christensen B, Svart M, Schmidt MS, Sulek K, Ringgaard S, Stodkilde-Jorgensen H, Moller N, Brenner C, Treebak JT et al. A randomized placebo-controlled clinical trial of nicotinamide riboside in obese men: safety, insulin-sensitivity, and lipid-mobilizing effects. Am J Clin Nutr. 2018;108(2):343–53. 29992272 10.1093/ajcn/nqy132

[9] Remie CME, Roumans KHM, Moonen MPB, Connell NJ, Havekes B, Mevenkamp J, Lindeboom L, De Wit VHW, van de Weijer T, Aarts SABM et al. Nicotinamide riboside supplementation alters body composition and skeletal muscle acetylcarnitine concentrations in healthy obese humans. Am J Clin Nutr. 2020;112(2):413–26. 10.1093/ajcn/nqaa072 PMC7398770 32320006

[10] Elhassan YS, Kluckova K, Fletcher RS, Schmidt MS, Garten A, Doig CL, Cartwright DM, Oakey L, Burley CV, Jenkinson N et al. Nicotinamide Riboside Augments the Aged Human Skeletal Muscle NAD^+^ Metabolome and Induces Transcriptomic and Anti-inflammatory Signatures. Cell Rep. 2019;28(7):1717–28. 31412242 10.1016/j.celrep.2019.07.043 PMC6702140

[11] Parker R, Schmidt MS, Cain O, Gunson B, Brenner C The NAD metabolome is functionally depressed in patients undergoing liver transplantation for alcohol-related liver disease. Hepatology Communications. 2020;4:doi:10.1002/hep4.1530. 10.1002/hep4.1530 PMC7395074 32766477

[12] Hamity MV, White SR, Walder RY, Schmidt MS, Brenner C, Hammond DL Nicotinamide riboside, a form of vitamin B3 and NAD+ precursor, relieves the nociceptive and aversive dimensions of paclitaxel-induced peripheral neuropathy in female rats. Pain. 2017;158(5):962–72. 28346814 10.1097/j.pain.0000000000000862

[13] Liu HW, Smith CB, Schmidt MS, Cambronne XA, Cohen MS, Migaud ME, Brenner C, Goodman RH Pharmacological bypass of NAD(+) salvage pathway protects neurons from chemotherapy-induced degeneration. Proc Natl Acad Sci USA. 2018;115(42):10654–9. 30257945 10.1073/pnas.1809392115 PMC6196523

[14] Trammell SA, Brenner C Targeted, LCMS-based metabolomics for quantitative measurement of NAD(+) metabolites. Comput Struct Biotechnol J. 2013;4:e201301012. 24688693 10.5936/csbj.201301012 PMC3962138

